# Characterizing neurolinguistic patterns of primary progressive aphasia and Alzheimer's disease in Arabic‐speaking patients: Case series

**DOI:** 10.1002/alz70857_100313

**Published:** 2025-12-24

**Authors:** Mohamed Taiebine, Mustapha El Alaoui Faris

**Affiliations:** ^1^ Euro‐Mediterranean University of Fez (UEMF), Fez, Fez, Morocco; ^2^ Alzheimer's center, Rabat, Morocco; ^3^ Faculty of Medicine and Pharmacy, University Mohammed V, Rabat, Morocco

## Abstract

**Background:**

Primary progressive aphasias (PPA) and Alzheimer's disease (AD) are a group of neurodegenerative diseases defined by a gradual decline of linguistic and neuro‐cognitive abilities. PPA are traditionally classified into four predominant variants: nonfluent‐agrammatic (PPA‐NAv, semantic (PPA‐Sv), logopenic (PPA‐Lv) and mixed (PPA‐Mv). We aimed at phenotyping PPA and AD in native speakers of Moroccan Arabic.

**Method:**

The study included 20 Arabic‐speaking participants: 14 with Alzheimer's Disease (4 mild, 10 moderate‐severe) and 6 with PPA (2 PPA‐NAv; 1 with PPA‐Sv; 2 with PPA‐Lv and 1 with PPA‐Mv). Participants underwent neuropsychological assessments, including the Moroccan version of the Mini‐Linguistic State Examination (MLSE), to evaluate various aspects of language function. The study employed the Kussmaul's model (Behforuzi et al., 2013) for neurocognitive analysis.

**Result:**

According to the Kussmaul's connexionist model, a significant number of patients in this case series with AD may retain visual recognition units, which facilitate access to a relatively preserved semantic‐conceptual field (8‐F‐3). Consequently, the phenomenon of dysnomia is likely attributed to challenges in accessing the phonological lexicon (8‐G‐4). In contrast, individuals diagnosed with nfv‐PPA also exhibit dysnomia, which is likely the result of a disconnection among the semantic‐conceptual module, the fronto‐subcortical intentional system, the phonetic processor, and the output lexicon (3‐H‐4 or 7‐K‐3‐H‐4). Conversely, the patients with Lv‐PPA exhibited progression to PPA+ characterized by episodic and semantic memory impairments.

**Conclusion:**

This study offers a viewpoint on the neurolinguistic features of AD and PPA based on Kussmaul's model. It highlights the deterioration of syntactic abilities and semantics within MLSE, which contrasts with phonology and motor speech.

